# Prolonged grief symptomatology following violent loss: the mediating role of meaning

**DOI:** 10.1080/20008198.2018.1503522

**Published:** 2018-08-14

**Authors:** Evgenia Milman, Robert A. Neimeyer, Marilyn Fitzpatrick, Christopher J. MacKinnon, Krista R. Muis, S. Robin Cohen

**Affiliations:** a Department of Educational and Counselling Psychology, McGill University, Montreal, QC, Canada; b Department of Psychology, Memphis University, Memphis, TN, USA; c Department of Oncology, McGill University, Montreal, QC, Canada; d Departments of Oncology and Medicine, McGill University, Palliative Care Research, Jewish General Hospital, Montreal, QC, Canada

**Keywords:** Prolonged Grief Disorder, mediation of bereavement outcome, meaning making, violent death, suicide, loss, 延长哀伤障碍, 对丧亲结果的中介, 赋予意义, 暴力死亡, 自杀, 丧亲, • Violent loss predicted higher levels of Prolonged Grief Disorder (PGD) symptomology by disrupting meaning making.• Specific themes of meaning found to be associated with PGD symptoms following violent loss include ‘continuing bonds’ and ‘sense of peace’.• ‘Continuing bonds’ represents meaning related to the symbolic relationship between the bereft and the deceased.• ‘Sense of peace’ represents meaning related to how the bereaved perceives the death event.

## Abstract

**Background:** Prolonged Grief Disorder (PGD) is over-represented among those who have lost loved ones to violent causes. To tailor PGD interventions for this vulnerable population it is critical to examine the aetiology of PGD specifically in the context of violent death bereavement. Previous studies have suggested that violent loss increases symptoms of PGD by hindering the mourner’s ability to make meaning of the death or its aftermath. However, these studies have relied on cross sectional data that preclude genuine prediction and have not differentiated among specific themes of meaning.

**Objective:** This study aimed to identify specific themes of meaning that mediate the detrimental impact of violent loss on subsequent emergence of PGD symptomatology among the violently bereft.

**Method:** A longitudinal, prospective design (*N* = 171) was used to assess violent loss and themes of meaning an average of six months post-loss allowing for prediction of PGD symptoms an average of eight months later.

**Results:** Violent loss had a significant indirect effect on PGD symptomatology when meaning themes focusing on sense of peace and continuing bonds served as mediators.

**Conclusions:** This study demonstrates the mediating role that specific meaning themes play in the development of PGD symptomatology following violent loss. These findings highlight the potential benefits of applying a meaning-based intervention approach with the violently bereft.

## La sintomatología del duelo^⋆^(grief) prolongado después de una pérdida violenta: El rol mediador del significado

**Antecedentes:** El Trastorno de Duelo Prolongado (PGD en su sigla en inglés) está sobrerrepresentado entre aquellos que han perdido a sus seres queridos por causas violentas. Para adaptar las intervenciones de PGD para esta población vulnerable, es fundamental examinar la etiología del PGD, específicamente en el contexto de la experiencia de pérdida (bereavement) violenta. Estudios previos han sugerido que la pérdida violenta aumenta los síntomas del PGD al obstaculizar la capacidad del doliente (mourner) de dar sentido a la muerte o sus consecuencias. Sin embargo, estos estudios se han basado en datos transversales que impiden una predicción genuina y no han diferenciado entre temas específicos de significado.

**Objetivo:** Este estudio tuvo como objetivo identificar los temas específicos de significado que median el impacto perjudicial de la pérdida violenta en la aparición posterior de la sintomatología PGD entre los dolientes.

**Método:** Se utilizó un diseño prospectivo longitudinal (*N* = 171) para evaluar la pérdida violenta y los temas de significado, aproximadamente 6 meses después de la pérdida, lo que permitió la predicción de los síntomas de PGD en un promedio de 8 meses después.

**Resultados:** La pérdida violenta tuvo un efecto indirecto significativo en la sintomatología del PGD cuando los temas de significado que se centran en la sensación de paz y continuidad de los vínculos/lazos sirvieron como mediadores.

**Conclusiones:** El estudio demuestra el rol mediador que desempeñan los temas de específicos de significado en el desarrollo de la sintomatología del PGD después de la pérdida violenta. Estos hallazgos resaltan los beneficios potenciales de aplicar un enfoque de intervención basado en el significado con los dolientes.

## Abstract (zh)

**背景**：在因暴力失去亲近者的事件中过于常见延长哀伤障碍（PGD）。为了让PGD干预措施适合这类脆弱人群，考察在暴力事件丧亲的研究下PGD的病因学至关重要。以前的研究表明，暴力事件丧亲会减弱哀悼者对死亡或其后果赋予意义的能力，从而增加PGD症状。然而，这些研究依赖于横截面数据，排除了真正的预测因素，并忽略了对特定的意义主题进行区分。

**目的**：本研究旨在识别，特定的意义主题在暴力事件丧亲对随后出现的PGD症状的负面影响的中介效应。

**方法**：使用纵向前瞻性研究设计（*N* = 171），对暴力事件丧亲和丧亲后平均6个月的意义主题进行评估，并用以预测平均8个月后的PGD症状。

**结果**：暴力事件丧亲对PGD症状有显著的间接影响，两个意义主题（关注和平感和持续联结）有中介效应。

**结论**：该研究证明了特定的意义主题在暴力事件丧亲后PGD症状学发展中的中介作用。这些研究结果强调了采用基于意义的方法干预暴力事件丧亲的潜在益处。

The death of a loved one can be a profoundly challenging life event, however disproportionately high levels of distress have been documented among those grieving a loss due to homicide, suicide, or fatal accident – collectively referred to as violent bereavement (Kessler, Chiu, Demler, & Walters, 2005; McDevitt-Murphy, Neimeyer, Burke, Williams, & Lawson, 2012). For example, Prolonged Grief Disorder (PGD), which is a protracted, clinically significant, and functionally impairing form of grief, is experienced by 10–15% of those grieving a non-violent death (Lundorff, Holmgren, Zachariae, Farver-Vestergaard, & O’Connor, 2017; Prigerson et al., 2009) as compared with 30–70% of those grieving a violent death (McDevitt-Murphy et al., 2012; Mitchell, Kim, Prigerson, & Mortimer-Stephens, 2004; Momartin, Silove, Manicavasagar, & Steel, 2004; Shear, Jackson, Essock, Donahue, & Felton, 2006). PGD has been differentiated from Post-Traumatic Stress Disorder (PTSD) and post-loss depression (Boelen & Van Den Bout, 2005; Bonanno et al., 2007; Lichtenthal, Cruess, & Prigerson, 2004). It is referred to as Persistent Complex Bereavement Disorder under the section on conditions for further study in the DSM-5 (American Psychological Association, 2013), and will be introduced as a mental health diagnosis in the ICD-11 (World Health Organization, 2018; Maciejewski, Maercker, Boelen, & Prigerson, 2016). PGD also predicts a host of additional health problems, including heart disease, high blood pressure, cancer (Prigerson et al., 1997; Prigerson, Vanderwerker, & Maciejewski, 2008), suicidality (Latham & Prigerson, 2004), substance abuse, depression, anxiety, and overall life disruption (Boelen & Prigerson, 2007; Ott, 2003; Shear et al., 2011). Of note, although PGD constitutes an identifiable and distinct disorder, research indicates that it is best conceived of not as a qualitatively distinct taxon or category, but rather as an extreme form of protracted symptomatology that is on a continuum with normative grief responses (Holland, Neimeyer, Boelen, & Prigerson, 2009), and hence can be analysed as a continuous construct that varies in its severity. Given the striking proportion of violently bereft who experience the extensive health burden of PGD, it is vital to establish preventive interventions that are tailored for this exceptionally vulnerable population by examining the aetiology of PGD specifically in the context of a violent loss.

Studies have begun to identify the process by which poor mental health outcomes develop following violent loss (Boelen, De Keijser, & Smid, 2015; Mancini, Prati, & Black, 2011). For example, previous studies have suggested that risk factors for PGD, including that of violent loss, increase symptoms of PGD by hindering meaning making such that the bereft are unable to ‘make sense’ of the death or its aftermath (Milman, Neimeyer, Fitzpatrick, MacKinnon, Muis, & Cohen, under review; Currier, Holland, Coleman, & Neimeyer, 2007; Lichtenthal, Neimeyer, Currier, Roberts, & Jordan, 2013; Neimeyer, Baldwin, & Gillies, 2006; Rozalski, Holland, & Neimeyer, 2017). Building on this research, this study examines which themes of meaning are disrupted specifically following violent loss with the aim of informing preventive interventions that facilitate meaning making in a manner that mitigates symptoms of PGD among the violently bereft.

In accordance with its cognitive-constructivist roots, the meaning making paradigm suggests that individuals construct a system of orienting beliefs (Boelen, Van Den Hout, & Van Den Bout, 2006), self-narratives (Neimeyer, 2006b), personal constructs (Kelly, 1955), world assumptions (Janoff-Bulman, 1989) or schemas (Williams, Davis, & Millsap, 2002) about their identity and the world. This orienting system fosters a sense of meaning regarding the daily events individuals experience and their evolving life narrative (Kelly, 1955; Neimeyer, 2006a, 2016). However, death-related loss can challenge the orienting system of the bereft leading them to engage in meaning making (Park, 2010; Park & Folkman, 1997). This process entails retaining, reaffirming, revising, and/or replacing elements of the orienting system to develop more nuanced, complex, and useful narratives that accommodate the reality of the loss and its implications for the mourner (Bonanno, Wortman, & Nesse, 2004; Gillies, Neimeyer, & Milman, 2014; MacKinnon et al., 2013; Neimeyer, 2000; Neimeyer & Sands, 2011; Park, 2010; Steger, Frazier, Oishi, & Kaler, 2006). When the bereft make progress in negotiating the effect of the loss on their orienting systems, they have made meaning (Gillies et al., 2014). Thus, the term meaning has been used to refer both to the process of meaning making, wherein one finds/constructs meaning, as well as the outcome of this process, meaning made, wherein one has found/constructed meaning (e.g. Gillies et al., 2014). The manner in which meaning manifests is determined by how the bereaved have negotiated the challenge to their orienting systems. Thus, having made meaning is not necessarily expressed in statements regarding sense of purpose or meaning in life; instead, the content of meaning made varies. For example, if the bereaved is negotiating a sense of being lost in a world without the deceased, having made meaning might manifest in the development of a symbolic, guiding relationship with the deceased, otherwise referred to as a continuing bond. Alternatively, if the bereaved is struggling with the uncertain nature of life following a sudden loss, meaning made might entail a sense of valuing life despite this uncertainty. However, in cases where the meaning making process is hindered, such as following violent loss, the bereft remains in a distressing state of meaninglessness that impedes engagement with on-going life thereby fostering symptoms of PGD (Neimeyer, 2006a). Gillies, Neimeyer, and Milman (2015) carried out a content analysis of bereavement narratives identifying how meaning manifests in written accounts of the grief experience. The meaning themes that emerged were used to develop the Grief and Meaning Reconstruction Inventory (GMRI), which is comprised of five factors. The first factor, Emptiness and Meaninglessness (e.g. ‘I feel empty and lost’), captures themes of meaning that are negatively valenced in terms of the emotions that they engender. The remaining four factors reflect positively valenced meaning themes that represent the domains of the orienting system that are challenged by the death-event and, by extension, the domains within which meaning is made. These are as follows: relationship with the deceased represented by the continuing bonds subscale (e.g. ‘I cherish the memories of my loved one’), sense of self/identity represented by the personal growth subscale (e.g. ‘Since this loss, I’m more self-reflective’), perception of life represented by the valuing life subscale (e.g. ‘I value and appreciate life more’), and perception of the death represented by the sense of peace subscale (e.g. ‘This death brought my loved one peace’). Taken together, the GMRI subscales allow for a nuanced assessment of whether and what kind of meaning the bereaved has made of the loss experience.

The traumatic nature of violent loss lies in its distinctive death story, characterized by the grotesque quality of the death event, the transgression of the deceased’s will, and/or intentional action on the part of the perpetrator or the deceased (Rynearson & McCreery, 1993; Rynearson, Schut, & Stroebe, 2013). These unique features of the violent death story may impede meaning making as they challenge particularly core or fundamental orienting beliefs about the world as a safe and fair place where events are somewhat predictable and happen for a reason (Currier, Holland, & Neimeyer, 2009; Janoff-Bulman, 1989; Lichtenthal, Currier, Neimeyer, & Keesee, 2010). As result, there is an ongoing struggle to make meaning of the violent death story, often experienced in terms of intrusive negative thoughts or imagery related to the death event (Baddeley et al., 2015; Ehlers & Clark, 2000; Rheingold, Zinzow, Hawkins, Saunders, & Kilpatrick, 2012; Rynearson & Correa, 2008; Steil & Ehlers, 2000; Zinzow, Rheingold, Byczkiewicz, Saunders, & Kilpatrick, 2011). We propose that incongruence between the violent death story and the perception of the world as fair, predictable, and safe is likely to disrupt meaning associated with the sense of peace subscale of the GMRI. In other words, the violently bereft struggle to make meaning of the death as an event for which there was some preparation or which offered some release from suffering. Furthermore, aversive thoughts and imagery related to the death event may foster negatively-valenced meaning making resulting in higher scores on the emptiness and meaninglessness subscale of the GMRI. Finally, scholars have suggested that the cognitive association between the distressing nature of the violent death story and the mental representation of the deceased may cause the bereaved to avoid engaging in a meaningful symbolic relationship with the deceased (Currier, Irish, Neimeyer, & Foster, 2015; Meier, Carr, Currier, & Neimeyer, 2013; Rubin, Malkinson, & Witztum, 2012; Rynearson & Salloum, 2011), which could disrupt meaning making related to the continuing bonds subscale of the GMRI. Accordingly, this study tests the hypothesis that violent loss fosters higher levels of PGD symptomatology in the first two years of bereavement by disrupting meaning associated with the sense of peace and continuing bonds subscales of the GMRI while exacerbating negative appraisals associated with the emptiness and meaninglessness subscale.

Alongside evidence of the negative consequences of bereavement, there is increasing recognition that a loved one’s death can serve as a catalyst for positive bereavement outcomes, such as well-being and posttraumatic growth (Calhoun, Tedeschi, Cann, & Hanks, 2010; Currier, Holland, & Neimeyer, 2012; Currier, Mallot, Martinez, Sandy, & Neimeyer, 2013). Furthermore, scholars have suggested that having made positive meaning regarding one’s identity and ongoing life, measured respectively by the personal growth and valuing life subscales of the GMRI, facilitates these positive outcomes (Calhoun et al., 2010; Coleman & Neimeyer, 2010). Thus, in contrast to the remaining GMRI subscales, the personal growth and valuing life subscales are likely not relevant to negative bereavement outcomes and therefore are not expected to mediate the effects of violent loss on the development of PGD.

To test these hypotheses, a mediation analysis was conducted with data collected in a previous study that examined six PGD risk factors, including violent loss. Violent loss and themes of meaning made represented by the subscales of the GMRI were assessed in the early stages of grief (2–12 months post-loss; *M* = 6.25 months) allowing for prospective prediction of PGD symptoms 7–10 months (*M* = 8.39 months) later. By employing a longitudinal design to examine the aetiology of PGD specifically in the context of violent bereavement, the current extension of the Milman, Neimeyer, Fitzpatrick, MacKinnon, Muis, & Cohen, (under review) study provides a nuanced assessment of how disruption of specific meaning themes influences the severity of the violent loss experience. Ultimately, such research could provide guidance on how preventive interventions can be tailored to target meaning making in a manner that could effectively mitigate PGD following violent loss.

## 1. Method

### 1.1. Procedure

The research protocol was approved by the appropriate institutional review boards. Online or paper-and-pencil surveys were completed by individuals 18 years or older and bereaved for 2–12 months. Participants were instructed to complete surveys in a private location of their choice at any time of their choosing. Informed consent was obtained with a written consent form that preceded the survey. Participants were assessed twice: within 2–12 months of loss (T1) and 7–10 months later (T2). At T1, the following variables were assessed: (1) predictor variable: violent loss and (2) mediating variables: each of the five meaning themes assessed by the five factors of the GMRI, including continuing bonds, valuing life, personal growth, sense of peace, as well as emptiness and meaninglessness. At T2, the dependent variable, symptoms of PGD, was assessed.

Recruitment procedures varied according to recruitment site in accordance with local requirements (Preliminary analyses indicated that it was appropriate to combine subsamples obtained through the various recruitment sites employed in this study. These results can be made available by contacting the first author). Family members of deceased patients from a palliative care unit at a Canadian urban hospital who had previously consented to be contacted regarding research participation were recruited via phone by trained research assistants. Undergraduate psychology students at an urban university in the USA received optional course credit for participation in the study. The study was posted on the university’s webpage which listed all research projects available for optional class credit. A multi-site grief service organization in the UK announced the study via its organizational Facebook and Twitter accounts and a grief-related website (e.g. grief-related blogging, online chat) posted descriptions of the study. The study was also posted on Amazon Mechanical Turk, which is an online platform that enables researchers to reimburse individuals for their anonymous participation in their research studies. Participants recruited via Amazon Mechanical Turk were reimbursed 3 USD for completing the survey at the first time-point and 2 USD for completing the survey at the second time-point. All above-listed online descriptions, postings, and announcements detailed the study’s inclusion criteria and offered a link to the online survey. Finally, a hospice in the USA sent letters describing the study and survey packages with pre-addressed, stamped return envelopes to family members of deceased patients who met the inclusion criteria for the study.

### 1.2. Characteristics of sample

A total of 357 participants completed the first data collection, and 171 (47.9%) of these participants also completed the second data collection. Univariate logistic regression analysis was employed to examine whether being lost to follow-up was related to demographic, predictor, or mediating variables. Participants who remained in the study were less likely to have experienced a violent loss (OR = 0.49, 95% CI [0.27, 0.91]), more likely to be older (OR 1.02, 95% CI: 1.01 to 1.03), and more likely to have lost a sibling (OR = 3.77, 95% CI [1.09, 13.03]) than participants who were lost to follow up. No other differences in sociodemographic, predictor, or mediator variables were identified between participants who completed the follow-up measurement and those who did not, both in the sample as a whole and specifically among those who experienced a violent loss.

The characteristics of the sample that completed both the first and second data collection are summarized in Table 1. Approximately one-third of the Time 2 sample reported losing a parent (*n* = 59, 34.5%), a spouse/partner (*n* = 36, 21.1%), or other relation (*n* = 61, 35.7%). At time 2, the mean score on the PG-13 was 23.33 (*SD* = 10.28). A cutoff of 34 has been suggested as an indicator of clinically significant PGD symptomatology. This score corresponds with the degree of symptom severity at one standard deviation above the mean in previous studies that employed the PG-13 (W. G. Lichtenthal, personal communication, 10 November 2017). Similarly, in this study, a score of 33.61 on the PG-13 was one standard deviation above the sample mean. The majority of the sample were women (*n* = 123, 72%) and participants ranged in age from 18 to 90 years (*M* = 44.3). Participants averaged just over six months post-loss at Time 1. Only one participant (0.6%) reported not completing high school, while the majority of participants completed postsecondary degrees (*n* = 132, 77.2%). Nearly 90% (*n* = 151) of participants experienced a natural death loss; among those participants who experienced a violent loss only accidental and suicide loss was reported.

**Table 1.** Characteristics of the sample.

Variable		*n* or *M*	% or *SD*
Time Since Loss (months)		6.25	2.36
Severity of PGD^a^		23.33	10.27
Age (years)		44.30	16.07
Gender	Female	123	71.9%
	Male	47	27.5%
Education	Less than High School	1	0.6%
	High School	35	20.5%
	College/Undergraduate Degree	92	53.8%
	Graduate Degree	31	18.1%
	Professional Degree	9	5.3%
Relationship to the Deceased	Grandparent	22	12.9%
	Spouse	36	21.1%
	Child	6	3.5%
	Sibling	12	7%
	Parent	59	34.5%
	Friend	18	10.5%
	Ex-Spouse/Partner	3	1.8%
	Other	14	8.2%
Cause of Death	Suicide	12	7.0%
	Accident	7	4.1%
	Illness	109	63.7%
	Natural Sudden	35	20.5%
	Other	7	4.1%

PGD = Prolonged Grief Disorder.

^a^ Severity of PGD was measured by participants’ score on the Prolonged Grief Disorder-13.

### 1.3. Measures

#### 1.3.1. Violent death

Violent death was measured using a multiple-choice item inquiring as to the cause of death. In accordance with the description of violent death established in the literature (Rynearson et al., 2013), responses were then coded either as natural death (i.e. sudden loss, such as death due to heart attack; anticipated loss, such as death due to fatal illness) or as violent death (i.e. death resulting from an accident, suicide, or homicide).

#### 1.3.2. Themes of meaning made

The GMRI (Gillies et al., 2015) is a 29-item, Likert-type (1 = Strongly Disagree to 5 = Strongly Agree), self-report measure which was developed with a bereaved sample and contains five factors each reflecting a theme of meaning made: Emptiness and Meaninglessness (e.g. ‘I feel empty and lost’), Personal Growth (e.g. ‘Since this loss, I’m a more responsible person’), Sense of Peace (e.g. ‘This death brought my loved one peace’), Continuing Bonds (e.g. ‘I cherish the memories of my loved one’), and Valuing Life (e.g. ‘I value and appreciate life more’). While the final four factors represent positive meaning themes that emerge when an individual has made adaptive meaning of the grief experience, items pertaining to the first factor are endorsed by those who have made negative meaning of their grief. Thus, responses to items associated with the first factor are reverse-keyed when scoring the GMRI, so that a higher score reflects less negative meaning. The items of the GMRI were developed by conducting a thematic analysis of participants’ narrative responses to one-item measures of meaning employed in previous research such as ‘How much sense would you say have you made of the loss? Please comment how’. At the time that the study was conducted there were no grief-specific, validated measures of meaning available in the literature, therefore concurrent validity could not be assessed. However, the GMRI has been found to prospectively predict levels of grief-specific distress, general psychological symptomatology, and functional impairments. The measure has also demonstrated adequate test-retest reliability and strong internal consistency. In the current sample, the GMRI demonstrated adequate to excellent internal consistency for the sense of peace subscale (α = 0.82), the personal growth subscale (α = 0.81), the emptiness and meaninglessness (α = 0.85), the continuing bonds subscale (α = 0.77), the valuing life subscale (α = 0.61), and the GMRI as a whole (α = 0.88).

#### 1.3.3. Prolonged Grief Disorder

The Prolonged Grief Disorder-13 (PG-13; Prigerson et al., 2009) is a self-report measure that consists of 13 items evaluating symptoms of PGD (e.g. ‘In the past month, how often have you felt yourself longing or yearning for the person you lost?’) on a 5-point Likert scale (1 = not at all to 5 = several times a day/overwhelmingly). The PG-13 has demonstrated predictive validity (Lichtenthal & Prigerson, 2015) and showed excellent internal consistency (α = 0.95) in the current sample.

### 1.4. Data analytic plan

Data analysis was conducted using SPSS version 23. Data were missing at random, and missing data did not exceed 5% on any survey item relevant to testing the hypotheses presented in this study. Data were deleted listwise. The sample size required to achieve power in a mediation/moderation analysis is 30 participants per parameter being estimated in a regression model (Hayes, 2013; Tabachnick & Fidell, 2013). Accordingly, in this study, the sample size required to detect an effect was 90 participants. After cases with missing data were removed, the sample size was sufficient to achieve the necessary power for each regression analysis; specifically, the analysis with lowest sample size after missing case removal was 152. Pearson correlation analysis was used to assess whether results are broadly consistent with theory and findings from existing research.

**Figure 1.**
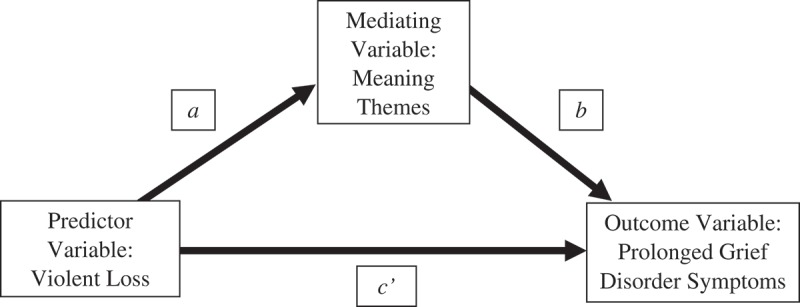
Conceptual diagram of the mediation hypotheses tested in this study. The relationship between violent loss and each theme of meaning made is represented by parameter *a*, the relationship between each meaning theme and PGD symptoms is represented by parameter *b*. The overall indirect effect is the product of the coefficient for *a* and the coefficient for *b* and is indicated by the notation *ab* throughout the results section. The direct effect of violent loss on PGD symptoms is represented by the path coefficient for parameter *c’*.

The mediation hypotheses represented by the conceptual diagram in Figure 1 were tested with ordinary least squares (OLS) regression analysis using SPSS add-on software entitled PROCESS. This data analysis procedure estimates path coefficients for the regression equations derived from the hypotheses depicted in Figure 1 (Hayes, 2013). Inferences regarding the presence of mediation are not based on the estimation of the individual paths that define the mediation pathway (parameters *a* and *b* in Figure 1), but rather are based on an estimate of the overall mediation pathway, also referred to as the indirect effect. The indirect effect is defined as a product of the individual regression coefficients that constitute the mediation pathway (see Figure 1 for more information). Although the presence of the hypothesized mediation pathways was inferred based on the significance of the indirect effect, individual path coefficients and their significance are reported to provide a comprehensive characterization of the individual relationships relevant to the study hypotheses.

Finally, the presence of a direct effect of violent loss on PGD symptoms was assessed by estimating the path coefficient for parameter *c’* (see Figure 1), which quantifies the degree to which violent death influences symptoms of PGD independent of its association with having made meaning.

## 2. Results

### 2.1. Bivariate correlations

Results of all bivariate correlations are summarized in Table 2. Consistent with previous research (Gillies et al., 2015) each factor of the GMRI demonstrated a significant, weak to moderately strong correlation with every other GMRI factor and with the total GMRI score. This finding is in line with the conceptualization of each GMRI factor as representative of a distinct theme of meaning made following loss.

**Table 2.** Correlations among Violent Loss, Meaning Made Themes, and Prolonged Grief Disorder.

Variable	1	2	3	4	5	6	7	8
1. Violent Death	__							
2. GMRI Emptiness and Meaninglessness	−0.03	__						
3. GMRI Sense of Peace	−0.26**	0.56**	__					
4. GMRI Continuing Bonds	−0.23**	0.19*	0.36**	__				
5. GMRI Valuing Life	0.06	0.23**	0.16*	0.49**	__			
6. GMRI Personal Growth	0.08	0.25**	0.19*	0.30**	0.67**	__		
7. GMRI Total Score	−0.13	0.72**	0.73**	0.65**	0.65**	0.66**	__	
8. PGD	−0.01	−0.67**	−0.48**	−0.17**	−0.25**	−0.21**	−0.57**	__

PGD = Prolonged Grief Disorder.

GMRI = Grief and Meaning Reconstruction Inventory.

*p < .05; **p < .01.

Contrary to previous research findings and theory (e.g. Burke & Neimeyer, 2013; Lobb et al., 2010), violent loss was not significantly correlated with PGD, the total GMRI score, the valuing life GMRI factor, the personal growth GMRI factor, or the emptiness and meaninglessness GMRI factor. However, violent loss was significantly correlated with the sense of peace and continuing bonds factors of the GMRI. Despite these findings, mediation analyses were carried out with each of the GMRI factors because an indirect relationship can be present in the absence of significant correlations pertaining to individual pairs of variables included in the indirect relationship (Hayes, 2013).

## 3. Mediation analyses

### 3.1. Indirect effects

Reviewing each individual relationship comprising the hypothesized mediation pathways (depicted in Figure 1), the results are as follows. Violent loss was associated with participants endorsing lower levels of the sense of peace meaning theme and the continuing bonds meaning theme (see path coefficients for parameter *a* in Table 3) and, in turn, participants who endorsed lower levels of either meaning theme reported significantly more PGD symptoms at the second data collection (see path coefficients for parameter *b* in Table 3).

**Table 3.** Regression coefficients for mediation pathways demonstrating a significant indirect effect.

	Model Predicting Meaning				Model Predicting Prolonged Grief Disorder			
Antecedent Variable	Pr	Coeff	SE	*p*	Pr	Coeff	SE	*p*
Violent Loss	*a*	−3.79**	1.27	.00	*c’*	−4.38	2.39	.07
Sense of Peace		___	___	___	*b*	−.99***	.15	< .001
Constant	i1	16.40***	.42	< .001	i2	39.64***	2.51	< .001
		R2 = .05** F(1,159) = 8.94, *p* = .00				R2 = .23*** F(2,158) = 22.99, *p* < .001		
Violent Loss	*a*	−3.85**	1.15	.001	*c’*	−2.07	2.69	.44
Continuing Bonds		___	___	___	*b*	−.37*	.18	< .001
Constant	i1	28.74***	.38	< .001	i2	34.06***	5.24	< .001
		R2 = .07** F(1,159) = 11.24, *p* = .001				R2 = .03 *F*(2,158) = 2.13, *p* = .12		

Pr = Parameter. Coeff = Regression Coefficient. *p < .05. **p < .01. ***p < .001.

Results also indicate a significant indirect effect of violent loss on the development of PGD symptoms based on 10,000 bootstrap samples when the sense of peace meaning theme served as a mediator (*ab* = 3.73, 95% CI: 1.68 to 6.34). The partially standardized indirect effect with sense of peace as a mediator is 0.36. This indicates that as a result of the disruption of the sense of peace meaning theme the violently bereft differ from the non-violently bereft, on average, by one-third of a standard deviation in symptoms of PGD. In addition, results indicate a significant indirect effect of violent loss on the development of PGD symptoms based on 10,000 bootstrap samples when the continuing bonds meaning theme served as a mediator (*ab* = 1.42, 95% CI: .16 to 3.91). The partially standardized direct effect with continuing bonds as a mediator is 0.14. This indicates that as a result of the disruption of the continuing bonds meaning theme the violently bereft differ from the non-violently bereft, on average, by one-sixth of a standard deviation in symptoms of PGD. By contrast, there was no significant indirect effect found when the valuing life (*ab* = – .52, 95% CI: −2.68 to .91), personal growth (*ab* = -.51, 95% CI: – 2.70 to .71), and emptiness and meaninglessness (*ab* = .59, 95% CI: −2.64 to 3.84) meaning themes served as mediators.

### 3.2. Direct effects

Results pertaining to the direct effect of violent loss can be found in Table 3 (see path coefficients for parameter *c’*). Violent loss did not demonstrate a direct effect in that it did not significantly predict symptoms of PGD independent of its influence on how much meaning the bereft made.

## 4. Discussion

Building on previous research that demonstrated the mediating role of meaning making in the aetiology of PGD (Milman, Neimeyer, Fitzpatrick, MacKinnon, Muis, & Cohen, under review; Currier, Holland, & Neimeyer, 2006; Rozalski et al., 2017), the present study is the first to identify themes of meaning that prospectively predict PGD symptomatology in the context of violent loss. Specifically, relative to non-violent bereavement, participants grieving a violent death endorsed sense of peace and continuing bonds to a lesser degree and, over time, those who reported lower levels of these meaning themes experienced higher PGD symptomatology.

These findings offer support for the hypothesis that the uniquely disturbing features of the violent death story – the grotesque quality of the death, violation of the deceased’s will, and/or voluntary action on the part of the perpetrator or the deceased (Rynearson & McCreery, 1993; Rynearson et al., 2013) – may prevent the bereft from making meaning of the death as an event for which there was some preparation or which offered some release from suffering (Currier et al., 2009; Currier, Holland, Coleman, & Neimeyer, 2008). Instead, research suggests that the bereft is confronted with actual or imagined scenes of the violent dying (Baddeley et al., 2015; Ehlers & Clark, 2000; Halligan, Clark, & Ehlers, 2002; Steil & Ehlers, 2000) that may become associated with and even overshadow memories of the deceased’s life (Rubin et al., 2012; Rynearson, 2005; Rynearson & McCreery, 1993). As a result, the bereft may engage in maladaptive attempts to minimize this distressing imagery by avoiding memories of the deceased, potentially impeding the construction of a meaningful, life-affirming, ongoing bond with the deceased. As suggested by the findings of this study, this struggle with the sense of peace and the continuing bonds meaning themes can impede the grieving process, fostering symptoms of PGD.

In order to address the detrimental impact of death imagery, cognitive-behavioural therapy (CBT) for PGD includes repeated imaginal exposure to death-related scenes as a means of facilitating tolerance of their distressing nature, thereby enabling the bereft to engage with and adequately process these scenes (Boelen, De Keijser, Van Den Hout, & Van Den Bout, 2007; Bryant et al., 2014; Shear, Frank, Houch, & Reynolds, 2005; Wagner, Knaevelsrud, & Maercker, 2007). In so doing, it may be that CBT interventions allow the bereft to access their innate meaning making capacities. Although these interventions have not been specifically developed for or tested with violent loss, they have demonstrated impressive outcomes.

Exposure-based techniques bolstering distress tolerance are also employed in meaning-oriented interventions, however in this context they are designed chiefly to facilitate meaning making regarding the death event. Specifically, the emotionally evocative details of the violent death story are reviewed in order to examine and address how it has challenged the orienting system of the bereft (Neimeyer, 2016; Neimeyer, Milman, & Steffen, in press; Rheingold et al., 2015; Rynearson & Correa, 2008; Rynearson & Salloum, 2011). This focus on the ‘event story’ of the death takes place in coordination with cognitive-constructivist (e.g. Neimeyer, 2012) and experiential (e.g. Milman, 2014) techniques that encourage the formation of a continuing bond by drawing on the ‘back story’ of the relationship with the deceased (Neimeyer, 2016; Neimeyer et al., in press). In other words, meaning-oriented interventions build distress tolerance as a precursor to a clinical process that targets meaning making related to both the death story and the relationship with the deceased. For example, after exploring a relationship that was marked by repeated struggles with the deceased’s uncontrolled mood disorder, a client might come to the sense that, despite the tragic nature of the suicide event that took the deceased’s life, the death itself represented a release from suffering for the deceased. Alternatively, the clinician might aid a client bereaved by a fatal vehicular accident in sifting through a relationship that was characterized by conflict and abuse related to the deceased’s substance use. Accordingly, the client might come to experience the death as bringing an end to suffering, both for the deceased and for the client.

Of note, CBT also describes a therapeutic process that encourages meaning making by identifying and revising how the death has impacted the mourner’s system of beliefs (Boelen et al., 2007; Bryant et al., 2014; Malkinson, 2007; Rosner, Pfoh, Kotoučová, & Hagl, 2014). Relative to meaning-oriented interventions, this cognitive restructuring process tends to be more goal-directed and is not necessarily designed specifically to foster a continuing bond with the deceased. Thus, both CBT and meaning-oriented clinical approaches appear to be well-suited for addressing the disruptive effects of violent loss on the sense of peace and/or continuing bonds meaning themes.

Violent bereavement is over-represented in marginalized sociodemographic populations, indicating the need for violent loss interventions to incorporate sources of resilience present in these populations (Bottomley, Burke, & Neimeyer, 2015; McDevitt-Murphy et al., 2012; Milman, Rheingold, Williams, & Bountress, in press). Meaning-oriented protocols make it possible to take advantage of such population-specific resiliency factors by exploring diverse sources of meaning (Neimeyer, 2016; Neimeyer et al., in press), including spirituality (Doka, 2012; Pearce & Smigelsky, 2015), artistic endeavours (Thompson & Neimeyer, 2014), family (Hooghe & Neimeyer, 2013), community (Murphy & Neimeyer, 2014; Smigelsky et al., 2016), and legacy work commemorating the deceased (Hochberg, 2012). This flexibility enables clinicians to foster a sense of peace regarding the death event and a continuing bond with the deceased in a manner that is compatible with the resources and protective factors that are accessible to diverse violently bereft populations. Accordingly, meaning-oriented interventions may be well-suited for mitigating PGD in the sociodemographic contexts where violent death tends to be over-represented. While preliminary research has shown promising outcomes with PGD (Neimeyer & Alves, 2017), violent loss (Rheingold et al., 2015; Saindon et al., 2014), and suicide bereavement (Supiano, Haynes, & Pond, 2017), randomized controlled trials testing the efficacy of the meaning-oriented approach with violent loss in marginalized populations are a high priority.

As expected, neither the personal growth nor the valuing life meaning themes mediated the effect of violent loss on PGD. However, this finding does not imply that these meaning themes are irrelevant to the violent bereavement experience. Instead, personal growth and valuing life may mediate positive bereavement outcomes. For example, post-traumatic growth is more likely to develop following violent than non-violent loss (e.g. Currier et al., 2012, 2013) and scholarship suggests that this association may be fostered by having made positive meaning regarding one’s identity and ongoing life (Calhoun et al., 2010), represented respectively by the personal growth and valuing life meaning themes in this study. Furthermore, the absence of a significant overall association between violent loss and PGD symptoms, despite the existence of a mediated effect, leaves open the possibility that the disruption of meaning is counteracted by a competing mediating mechanism that offsets rather than exacerbates symptoms of PGD following violent loss (Hayes, 2013). The construct of post-traumatic growth (PTG), which refers to positive change experienced as a result of a traumatic life event, could represent one such mechanism. Specifically, it may be that the opposing mediating pathways of increased PTG in the presence of decreased sense of peace and continuing bonds produce a negligible overall association between violent loss and PGD symptoms. Of note, there was no direct effect of violent loss on PGD symptoms, indicating that violent loss does not affect symptoms of PGD independent of a mediating pathway. While this finding emphasizes the primacy of mediating processes in the relationship between violent loss and PGD symptoms, it is possible that this study identified only one of multiple mediating processes. Thus, future research could effectively build on this study by examining the hypothesis that PTG mitigates the impact of violent loss on PGD. Furthermore, the personal growth and valuing life meaning themes might be examined as central constructs in this competing mechanism of action. Correlational analyses showed that the emptiness and meaningless theme is significantly associated with PGD symptoms in the sample overall. This finding is in line with the conceptual overlap between meaninglessness and PGD, wherein a sense of meaninglessness represents a potential symptom of PGD that is assessed by the PGD symptom measure employed in this study. However, despite this conceptual overlap and contrary to the hypothesis presented in this study, emptiness and meaninglessness did not mediate the development of PGD specifically following violent bereavement. The emptiness and meaningless theme captures meaning that is negatively valenced with items such as ‘I feel empty and lost’ or ‘Since this loss, I find myself alone and isolated’. It may be that this type of negative meaning is a common experience in the aftermath of loss in general, including loss by natural death. As a result, emptiness and meaninglessness might not be uniquely predictive of PGD following violent loss. Alternatively, it may be that negative meaning becomes maladaptive among mourners who have trait-level dispositions toward negative cognition, such as those who demonstrate high levels of neuroticism and anxious attachment. In fact, both of these traits have received considerable empirical support as risk factors for PGD (Burke & Neimeyer, 2013; Lobb et al., 2010). Thus, the emptiness and meaninglessness theme may be relevant to poor adaptation following violent loss if the griever demonstrates anxious attachment and/or neuroticism. Indeed, scholarship suggests that risk factors are likely to moderate each other’s effect on the grief experience in a manner that exacerbates symptoms of PGD beyond the influence of any one risk factor (Burke & Neimeyer, 2013; Lobb et al., 2010; Stroebe, Folkman, Hansson, & Schut, 2006). Future studies examining the aetiology of PGD would benefit from testing the empirical validity of these various scenarios.

When interpreting this study’s findings, several limitations must be considered. Although the study’s sample was diverse in terms of age and relationship to the deceased, the majority of participants were well educated and none of the participants experienced loss due to homicide. In addition, participants who completed the first and second data collection were less likely to have experienced a violent loss and more likely to have been older or to have lost a sibling, relative to participants who only completed the first data collection. These sample characteristics could have introduced bias to the findings. For example, accident, suicide, and homicide bereavement have distinct features, so each form of violent bereavement likely makes a unique contribution to the experiences of the violent loss population. Thus, the absence of homicide bereft participants in the violent loss sample may have decreased the representativeness of the study’s findings. In addition, the low proportion of violently bereft participants in the sample may have affected the accuracy of the parameter estimates obtained for the tested mediation models. Finally, this study did not control for the severity of PGD symptoms at the first data collection and, therefore, did not account for a potential relationship between meaning and grief severity in the early stages of bereavement. Thus, it is possible that meaning made mediates PGD symptoms not only over time, as this study suggests, but also acutely or contemporaneously. Alternatively, it may be that acute grief severity impacts both degree of meaning made and the persistence of PGD symptoms over time. Yet another possibility is that the mediational relationship between violent loss, meaning making, and grief severity includes a positive feedback mechanism, so that disrupted meaning and severe grief exacerbate each other throughout the course of bereavement, beginning in the early stages. Prospective longitudinal research commencing at the time of the death with a larger violent loss sample that includes homicide bereavement and more than two data-collection points would address these various limitations, providing a more detailed description of how meaning making and grief severity relate to one another as the experience of violent death bereavement progresses.

Despite these limitations, the study’s findings are bolstered by its convergence with cross-sectional research examining the mediating role of meaning in the association between violent death and PGD as well as by its longitudinal design and nuanced assessment of meaning. Consequently, the study provides initial evidence of the mediating role that the sense of peace and continuing bonds meaning themes play in the development of PGD symptomatology following violent loss. These findings highlight the potential benefits of applying a meaning-based intervention approach with the violently bereft. Further research is necessary to examine the clinical relevance of additional meaning themes both in terms of their detrimental interaction with risk factors for PGD and their relationship with positive bereavement outcomes. By providing a foundation for such research, this study can contribute to the design of preventive interventions that target the mechanisms driving the uniquely impairing nature of grief following violent loss.
